# Differential Contribution of Hypothalamic MAPK Activity to Anxiety-Like Behaviour in Virgin and Lactating Rats

**DOI:** 10.1371/journal.pone.0037060

**Published:** 2012-05-17

**Authors:** Benjamin Jurek, David A. Slattery, Rodrigue Maloumby, Katharina Hillerer, Sophie Koszinowski, Inga D. Neumann, Erwin H. van den Burg

**Affiliations:** Department of Behavioural and Molecular Neurobiology, University of Regensburg, Regensburg, Germany; Florida State University, United States of America

## Abstract

The c-Raf – MEK1/2 – ERK1/2 mitogen-activated protein kinase (MAPK) intracellular signalling cascade in neurons plays important roles in the control of a variety of behaviours, including social behaviours and anxiety. These roles partially overlap with those described for oxytocin (OXT), and it has been shown that OXT activates the MAPK pathway in the hypothalamus (of male), and hippocampus (of female) rats. Here, by combining behavioural (light/dark box) and biochemical analyses (western blotting), we tested two hypotheses: (i) that OXT is anxiolytic within the hypothalamus of females, and (ii) that this effect, as well as that of lactation-associated anxiolysis, depends on the recruitment of the MAPK pathway. We found that, when injected bilaterally into the hypothalamic paraventricular nucleus (PVN), OXT decreased anxiety-like behaviour in virgins, and that this effect depended on phosphorylation of MEK1/2. MAPK pathway activation in lactation was evident by high phosphorylated (p) MEK1/2 levels, and nuclear translocation of ERK1. The high pMEK1/2 levels were necessary for the anxiolytic phenotype typically observed during lactation. Interestingly, exogenous OXT in lactating rats reduced pMEK1/2 levels without a concomitant effect on anxiety, indicating that OXT receptor activation can lead to recruitment of additional intracellular pathways to modulate MEK activity. Still other pathways could include MEK, but without subsequent activation of ERK, as we did not observe any increase in OXT-induced ERK phosphorylation. Together the results demonstrate that the MAPK pathway, especially MEK1/2, is critically involved in the regulation of anxiety-like behaviour in female rats.

## Introduction

The c-Raf – MEK1/2 – ERK1/2 mitogen-activated protein kinase (MAPK) pathway is one of the most important and best-studied intracellular signalling pathways. This pathway conveys extracellular signals to intracellular effectors via activation of a variety of cell membrane receptors, and hence is responsible for a battery of effects. In the brain, extracellular signal-regulated kinases (ERK) are strongly activated by synaptic stimulation, and are essential for the induction and maintenance of synaptic plasticity that is thought to underlie memory and learning [1]. Furthermore, ERKs have been shown to regulate anxiety-like behaviour and to contribute to the control of social behaviours, including social memory and aggression, particularly via ERK2 [2]. These effects overlap, at least partially, with those reported for the neuropeptide oxytocin (OXT), and indeed it has been shown that OXT activates the MAPK pathway within the hypothalamic paraventricular nucleus (PVN) via transactivation of the epidermal growth factor receptor (EGFR) to regulate anxiety in male rats [3]. Interestingly, the related nonapeptide, arginine vasopressin (AVP), has been shown to activate the MAPK pathway *in vitro* [4] but did not alter its activity within the PVN when applied *in vivo* [3]. Further, it has been repeatedly demonstrated that exogenous central administration of AVP has an anxiogenic effect [5] and that its expression level within the PVN negatively correlates with anxiety-related behaviour [6], [7].

In females, the brain OXT system is particularly active during the peripartum period with elevated rates of synthesis of the neuropeptide and its receptor, enhanced local release, and receptor binding within limbic and hypothalamic regions (for review see [8], [9], [10]). Such high brain OXT levels are important to induce anxiolysis during lactation [11], [12]. Also, OXT enhances spatial memory in the hippocampus of lactating rats, thought to improve the recollection of locations where food and water are present, and thus to reduce the time the mother needs to spend finding resources and leaving the pups unattended [13]. This effect of OXT on spatial memory depends on the activation of ERK, and one of its downstream effectors, the CRE responsive element binding protein (CREB) [13]. This example, together with the anxiolytic effect described above in males, shows the importance of ERK and its kinase MEK as intracellular mediators of the behavioural effects of OXT signalling. Further, it shows that the MAPK signalling pathway is recruited during lactation in the hippocampus, and that its recruitment depends on OXT. Moreover, given lactation-associated anxiolysis, and the role of both OXT and MAPK in anxiety, this pathway may be necessary for this effect. However, the roles of MEK and ERK in the PVN of females as effectors of anxiety-related behaviour have not been reported to date. This is nevertheless of particular importance considering the mood changes that frequently occur at peripartum in humans ([9] and references therein), and the reported pro-social and anxiolytic effects of OXT in males and females, in rats as well as in humans [3], [5], [14], [15], [16], [17].

**Figure 1:**
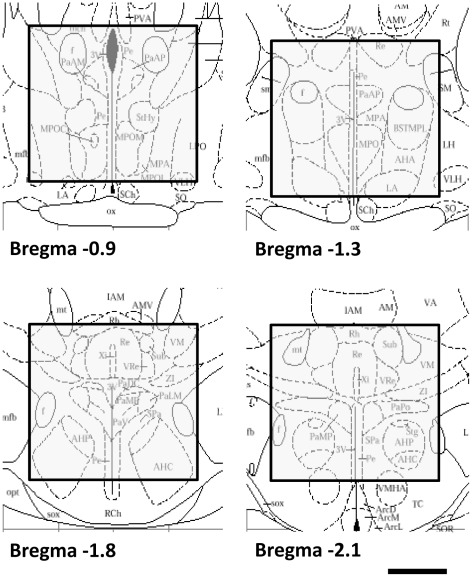
Schematic representation of PVN-enriched hypothalamic tissue that was extracted for western blot analyses. Scale bar: 1 mm.

Therefore, the present paper is concerned with the central question of whether MAPK pathway activity within the PVN of female rats is necessary for an anxiolytic phenotype. To address this, we employed two distinct approaches: (1) acute pharmacologically-induced (i.e. application of exogenous OXT), and (2) long-term physiologically-induced (i.e. lactation) anxiolysis and assessed their effects on the MAPK pathway within the PVN (Figure 1).

## Results

### Experiment 1. Effect of intra-PVN infusion of OXT on anxiety-related behaviour in virgin and lactating rats: effects of pre-treatment with the MEK1/2 inhibitor, U0126

**Figure 2:**
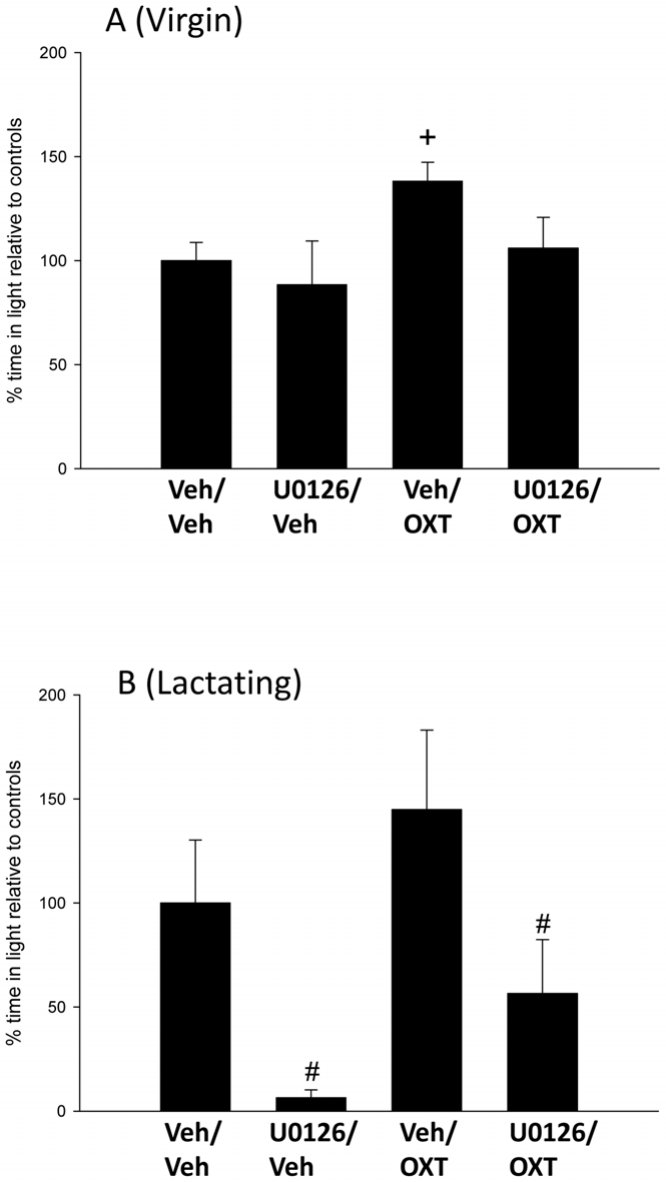
The effects of acute bilateral PVN administration of vehicle (Ringer solution, pH 7.4; Veh), or OXT (0.01 nmol/0.5 ul) after intra-PVN pre-treatment with either vehicle (DMSO) or the MEK 1/2 inhibitor, U0126 (0.5 nmol/0.5 ul) on time spent in the light compartment of the LDB in (A) virgin rats and (B) lactation day 8 rat dams. Data represent mean + sem (n = 7–12 per group). Two-way ANOVA was performed followed by Fisher's LSD *post-hoc* tests where appropriate. * p<0.05 compared with vehicle treatment and # p<0.05 compared with respective virgin group.

In virgin rats, ANOVA did not reveal a significant effect of either bilateral infusion of U0126 (F_1,29_ = 2.21; p = 0.15) or OXT (F_1, 29_ = 3.59; p = 0.068) directly into the PVN on the relative time spent in the light box of the light dark box (LDB). However, separate analyses revealed a significant anxiolytic effect of vehicle/OXT infusion (MWU; p = 0.009 versus vehicle/vehicle group; Fig. 2A) while there were no significant differences between the three other groups. This effect was not paralleled by altered locomotor activity as the number of line-crosses in the dark compartment did not differ between the treatment groups (data not shown).

In contrast to the effect observed in virgins, a significant effect of MEK1/2 inhibition on anxiety was observed in lactating rats (F_1,32_ = 11.3, p = 0.002). Specifically, U0126 treatment increased basal anxiety in lactating rats (p<0.05 versus vehicle/vehicle, Fig. 2B) without altering locomotor activity (data not shown). Interestingly, bilateral intra-PVN infusion of synthetic OXT did not affect anxiety in lactation (F_1,32_ = 3.08; p = 0.089; Fig. 2B).

### Experiment 2. Determination of basal and OXT-induced MEK 1/2 and ERK1/2 activation within the PVN in virgin, pregnant, and lactating rats

**Figure 3:**
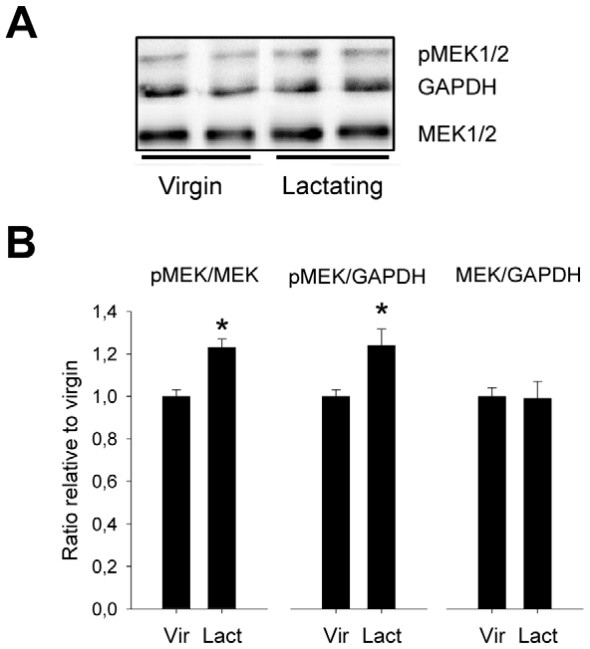
MEK phosphorylation as function of reproductive status in female rats. A, Representative blot of cytoplasmatic pMEK and MEK from PVN protein extracts from virgin and lactating rats. B, Cytoplasmatic pMEK levels were higher in the PVN of lactating rats when compared with total MEK and total protein (as measured by GAPDH amount), whereas total MEK levels were similar in both groups. Data relative to virgin control value of 1. Mann-Whitney *U*-test, * p<0.05.

To determine whether reproductive status altered hypothalamic MEK1/2 activation within the PVN, we analysed phosphorylated MEK1/2 (pMEK1/2) levels relative to total MEK and protein content in virgin and lactating (LD8) rats. pMEK1/2 levels appeared to be 24±7% higher in the cytoplasmatic fraction of lactating rats than in virgin rats (p = 0.04), and this applied to both the pMEK/MEK and the pMEK/GAPDH ratio (Fig. 3). The MEK/GAPDH ratio was similar in both groups, indicating that the increased pMEK1/2 concentration is due to increased phosphorylation, rather than to increased MEK synthesis, during lactation. There were no effects of reproductive state on nuclear MEK phosphorylation or content (data not shown).

**Figure 4:**
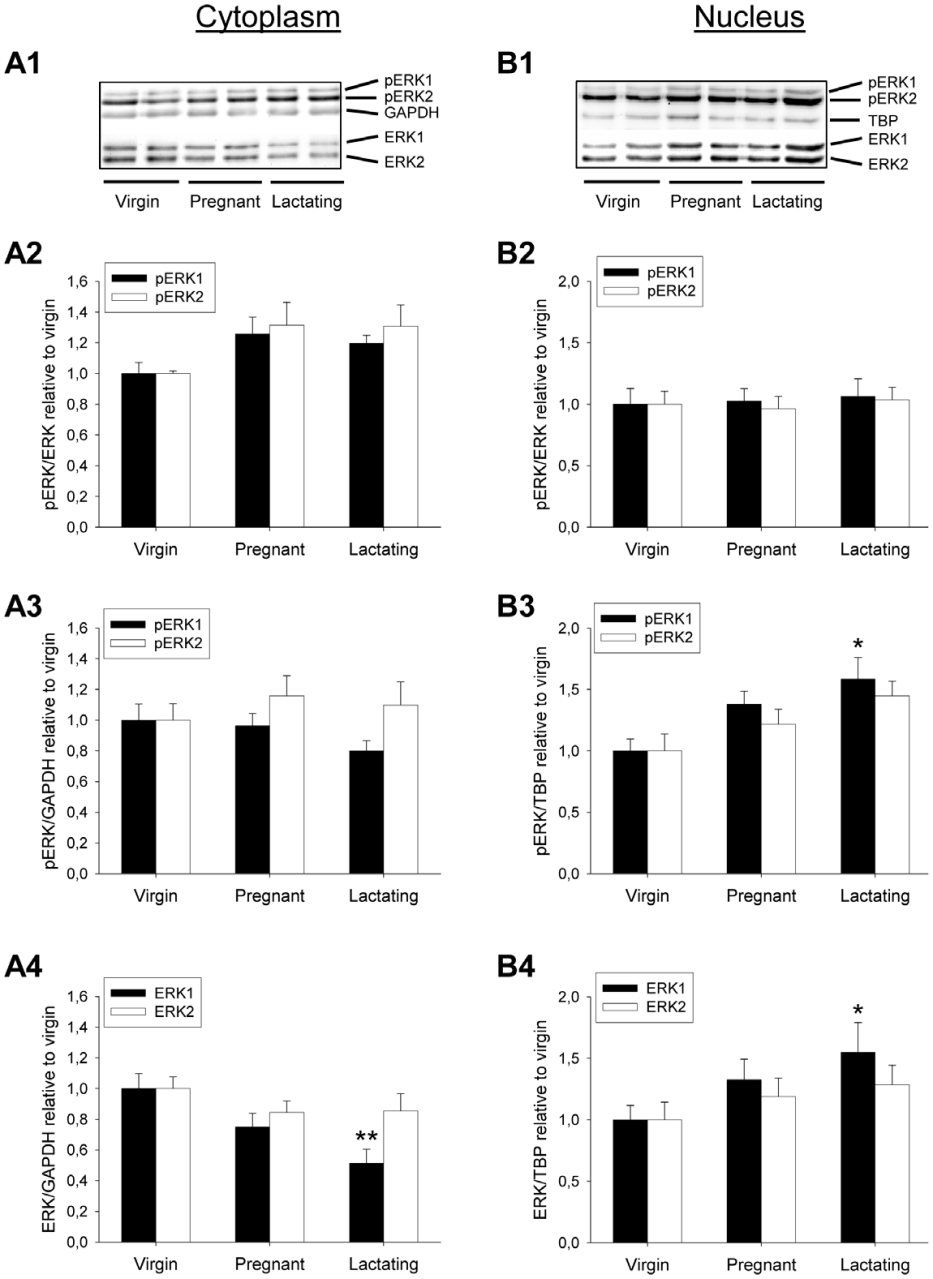
ERK phosphorylation status in cytoplasmatic (A) and nuclear (B) protein fractions from PVNs of virgin, pregnant, and lactating rats. A1, B1; representative cytoplasmatic and nuclear blots. A2, ERK phosphorylation in the cytoplasm tended to increase during pregnancy and lactation when corrected for the amount of total kinase, although not significantly. A3, cytoplasmatic pERK levels did not change during peripartum, when corrected for total protein amount (as measured by GAPDH levels). A4, Cytoplasmatic ERK1 concentration decreased during lactation, when compared with total protein amount. B2, Nuclear pERK/ERK ratio is constant over the peripartum period. B3, pERK1 levels were elevated in the nuclear fraction of lactating animals, when corrected for total amount of protein (as measured by TBP levels). B4, Nuclear total ERK1 levels were elevated in lactating rats. ANOVA was performed followed by Tukey's and Bonferroni's *post-hoc* tests where appropriate. All data are relative to virgin control values (set to 1). * p<0.05 and ** p<0.01 compared with respective virgin group. Vir, virgin; Lact, lactating.

Cytoplasmatic pERK1, but not pERK2, levels relative to both loading controls (GAPDH and β-tubulin; data not shown) tended to decrease in late-pregnant rats, and this effect reached significance in lactating rats (to 51.4% relative to virgin; F_2,17_ = 6.65; p = 0.009; Fig. 4A). In parallel, the nuclear pERK1 content increased in lactating rats as measured relative to the TATA box binding protein (TBP) loading control (1.6-fold increase relative to levels in virgins; F_2,17_ = 5.68; p = 0.016; Fig. 4B3). This was accompanied by a similar increase of the ERK1/TBP ratio (1.5-fold increase; F_2,17_ = 4.17; p = 0.04; Fig. 4B4). The ratio of pERK1/ERK1 was, in contrast, constant in both cellular compartments (Figs. 4A2, 4B2), indicating that in lactating rats, but not in late-pregnant rats, ERK1 is phosphorylated, and subsequently translocates to the nucleus.

**Figure 5:**
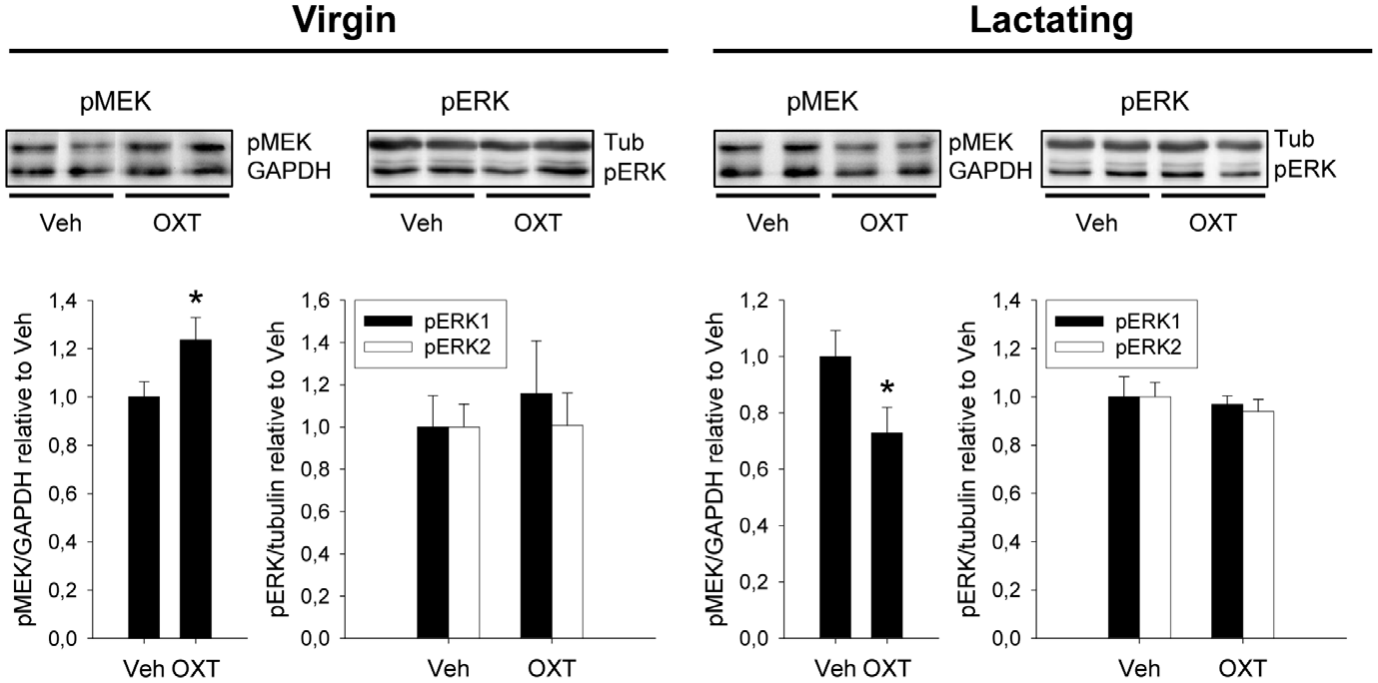
The effect of acute icv administration of oxytocin (OXT) on MEK and ERK phosphorylation in the cytoplasmatic fraction of PVN tissue of virgin and lactating rats. In virgins, OXT induced the phosphorylation of MEK, but not ERK, while in lactating rats OXT induced dephosphorylation of MEK, without affecting pERK levels. Total MEK and ERK levels did not change (not shown). Data are relative to vehicle (Veh) control groups for each reproductive state. Mann-Whitney *U*-test, * p<0.05.

Infusion of OXT (1 nmol icv) increased pMEK levels, relative to total MEK and protein content, by 24±9% in the cytoplasmatic fraction of PVN tissue of virgin rats (p = 0.049; Fig. 5). Surprisingly, MEK activation was not accompanied by increased ERK1/2 phosphorylation in virgins (Fig. 5). In lactating rats, where brain OXT activity is already high, pMEK levels were decreased in response to icv OXT (−27±9%; p = 0.049 versus lactating, vehicle-treated rats; Fig. 5). Again, the icv OXT infusion did not alter pERK1/2 levels neither in the cytoplasm nor in the nucleus.

### Experiment 3. Effects of blockade of the MAP kinase pathway on basal and OXT-induced ERK1/2 kinase activation within the hypothalamus of virgin and lactating rats

**Figure 6:**
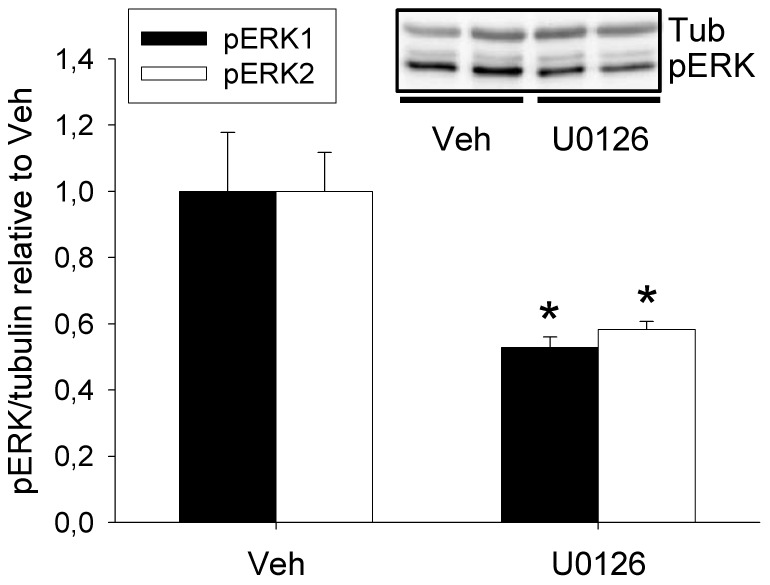
The effect of acute icv administration of U0126 on ERK1/2 phosphorylation in the cytoplasmatic fraction of the PVN of lactating rats. U0126 reduced phosphorylation of both ERK1 and ERK2; highly similar results were obtained in virgin animals (not shown). Data are expressed relative to respective vehicle (Veh) control group. Mann-Whitney *U*-test, * p<0.05. Tub, β-tubulin.

As the OXT-induced phosphorylation of MEK1/2 appeared to be uncoupled from the phosphorylation of ERK1/2, we inhibited the activity of pMEK1/2 pharmacologically with U0126 (1 nmol, icv) to determine whether pMEK1/2 exerts ERK1/2 phosphorylation activity in the PVN of female rats. Treatment with U0126 lowered basal pERK1/2 concentrations to a similar extent in both virgin and lactating rats (where pMEK1/2 had a profound effect on basal anxiety-like behaviour; Fig. 2B) to 52.9% (pERK1) and 58.4% (pERK2) of control (vehicle, DMSO-treated rats) levels (p = 0.019; Fig. 6), indicating that pMEK1/2 is indeed a kinase of ERK1/2.

## Discussion

In the present study we reveal that the complex neuronal adaptations, observed during lactation, include a tonic activation of MEK1/2 and ERK1 within the hypothalamic PVN. These effects were specific to lactation as no alterations in MAPK pathway activity in late pregnancy were observed. Acute local blockade of this MAPK signalling pathway in lactating rats caused a profound anxiogenic phenotype, indicating its involvement in lactation-associated anxiolysis. In addition, central OXT infusion resulted in activation of MEK1/2, as well as in anxiolysis in virgin rats, while in lactating rats OXT reduced, rather than increased, MEK1/2 phosphorylation without influencing anxiety-related behaviour. This suggests that in lactating rats, OXT might recruit additional pathways that could play a role in anxiolysis as endogenous levels are already high. Taken together, these results have implications regarding the mechanisms underlying the emotional alterations observed at peripartum, the reduced anxiety level, and increased calmness observed in breast-feeding women in particular.

The anxiolytic effect of endogenous brain OXT has been well-documented in female rats during the peripartum period [12], [18], [19]. A major site of synthesis and release of endogenous OXT is the PVN, and both aspects have been demonstrated to be up-regulated during lactation [8], [20], [21]. Here, we also demonstrate, for the first time, an anxiolytic effect of synthetic OXT directly within the PVN of virgin female rats. These results, together with our recent findings in male rats [3], [22], suggest that an increase in the availability of extracellular OXT within the PVN, either by local neuronal release or local infusion, results in anxiolysis independent of sex. The effects of synthetic OXT on anxiolysis were recently shown to be highly peptide-specific [3], and not due to actions at the receptor for the closely related neuropeptide vasopressin. Indeed, vasopressin has been consistently shown to increase anxiety [23].

The anxiolytic effect of intra-PVN OXT in virgin rats was abolished by prior administration of a MEK inhibitor, U0126, supporting recent findings in males [3], [24], [25]. This suggests that MEK mediates OXT-induced anxiolysis within the PVN in both male and female rats.

Our results presented here demonstrate that there is an activation of the MEK1/2 – ERK1 signalling cascade in the PVN during lactation. This was indicated by the profound anxiogenic effect of blockade of this pathway only in lactating rats, the increased pMEK1/2 levels in cytoplasmatic PVN extracts from mid-lactating rats compared with virgins, and by increased pERK1 and ERK1 levels in the nucleus; indicative of nuclear translocation. Our analysis of ERK phosphorylation and translocation, being the final results of MAPK activation, in both pregnant and lactating rats further showed that MAPK signalling only changes after giving birth; when the robust alterations in anxiety levels occur [9], [10]. Moreover, in contrast to virgin and male [3] rats, where acute OXT administration resulted in an increased pMEK/MEK ratio, OXT did not cause further activation of MEK1/2 in lactating rats, and was concomitantly without effect on anxiety-like behaviour. One might suggest that, as the endogenous OXT tone is already at a high level in the peripartum period [21], application of exogenous OXT does not elevate MEK activity further. Such a ceiling effect is likely not the only explanation, however, because OXT even *reduced* pMEK levels in lactating rats, indicating that OXT recruits one or more additional intracellular pathways in these animals leading to, amongst others, inhibition of MEK1/2. Although the tissue samples used for Western Blot analyses are PVN-enriched, and U0126 and/or OXT were infused into the PVN for the behavioural studies, we cannot completely rule out the possibility that the observed effects are partially mediated by surrounding hypothalamic regions, such as the neighbouring ventromedial hypothalamus. However, OXT infusions here have been shown to modulate sexual behaviour, rather then anxiety-related behaviour [26]. Taken together with the spatial restriction observed following small volume infusions into the brain [27], [28], this supports the conclusion that the role of hypothalamic MAPK activity in anxiety is predominantly localised within the PVN.

While MEK phosphorylation, as measured by western blotting, and MEK activity-dependent anxiety-like behaviour were in perfect agreement, ERK phosphorylation was negligibly influenced by icv OXT infusion. This is quite remarkable, as ERK1/2 are the only known targets of MEK1/2 in the brain to date. Interactions of MEK with its substrates (and upstream kinases) are organised by several scaffolding proteins including KSR [29], MP1 [30], and disc-large [31]. It seems reasonable to assume that MEK, being part of a large signalling complex anchored to scaffolding proteins, could physically interact with substrates other than ERK1/2 as well. Indeed, in human monocytes, a MEK2 – PI3Kδ pathway has been identified that operates independently from ERK, and serves to stimulate the production of an endogenous interleukin 1 receptor antagonist [32]. Other proteins that directly interact with MEK are the TGF-β receptor type II in a lymphoma cell line [33], and tumor suppressor WOXI in lysosomes of Jurkat cells (promotes apoptosis in T cell leukemia [34]). Also, MEK has been found to translocate to and from the nucleus, where it directly phosphorylates the transcription factor MyoD in differentiating myoblasts, thus influencing gene transcription [35]. Finally, MEK has been shown to bind the nuclear receptor peroxisome proliferator-activated receptor γ (PPARγ), then to export it out of the nucleus, and hence, to reduce PPARγ-controlled gene expression [36]. It is tempting to speculate that in PVN neurons several pMEK1/2 substrates exist as well, and that these are recruited following stimulation with OXT to bring about anxiolytic effects.

Although ERK1/2 phosphorylation appeared not to be controlled by OXT in the PVN of virgin and lactating female rats, our experiments with U0126, a blocker of pMEK1/2 activity, showed nevertheless that pMEK1/2 is involved in the control of “basal” (i.e. not stimulated with OXT) ERK1/2 phosphorylation. This shows that our protocols allow for the detection of subtle changes in ERK phosphorylation status, and that the lack of ERK phosphorylation in response to OXT treatment is not caused by methodological issues. In support of this, using the very same protocols as described here, we observed changes in pERK levels in the striatum of male rats that were exposed to social stimuli (Lukas, Neumann, Van den Burg, unpublished).

The only effect of reproduction on pERK we observed was the apparent translocation of pERK1 (but not pERK2) to the nucleus in lactating animals. We believe this accommodates the changes at the gene expression level necessary to induce neuroplasticity and stable rewiring of the neural circuitries that occur in lactation, resulting in physiological, psychological, and behavioural adaptations. Thus, it has consistently been reported that the PVN undergoes major morphological alterations during the peripartum period. This has been associated with increased OXT activity, both in the supraoptic nucleus and PVN [37], [38], [39]. The differential effect of reproductive status on ERK1 and ERK2 adds to the notion that these two closely related MAP kinases exert separate functions in the brain. It has for long been believed that ERK1 and ERK2 are redundant, but elegant experiments employing genetic strategies (such as the generation of knockdown, knockout, and conditional knock-out mice) have revealed especially ERK2-specific effects on learning and memory [40], as well as on the regulation of complex behaviour, including social behaviour [2]. Although ERK1 was reported not to influence learning and memory [41], a later study using ERK1 knockout mice revealed a specific role of ERK1 synaptic plasticity and drug addiction in the striatum [42]. Therefore, the translocation that we observed in lactating rats may, at least in part, underlie the plasticity seen within the PVN of lactating dams, in addition to the anxiolysis.

In conclusion, we have shown that the peripartum period in rats is accompanied by complex alterations in MEK – ERK signalling, which has important implications for anxiety-like behaviour. We favour a model in which both MEK1/2 and ERK1 are necessary to develop the anxiolytic phenotype observed during lactation (on the basis of increased MEK phosphorylation and ERK translocation in lactating animals). Some other MEK-controlled factor(s) must be responsible for the anxiolytic effect of OXT in virgin rats, because of lack of ERK1/2 phosphorylation. In contrast, in lactating rats there might be a switch in the recruitment of intracellular pathways coupled to the OXT receptor, as pMEK1/2 levels were down-regulated. Taken together, these results highlight the central importance of MEK for acute, OXT-induced, anxiolysis in virgins, and the maintenance of an anxiolytic phenotype during lactation.

## Materials and Methods

### Animals

Adult female Wistar rats (Charles River, Germany, 220–260 g body weight at the beginning of the experiment) were housed under standard laboratory conditions in groups of 3 to 4 (12 h light∶dark cycle, 22–24°C, lights on at 06.00 h, food and water ad libitum). All experiments were performed between 08:00–11:00, approved by the government of the Oberpfalz, Germany, and performed in accordance with the Guide for the Care and Use of Laboratory Animals by the National Institutes of Health, Bethesda, MD, USA.

After at least one week of habituation, mating with a sexually-experienced male (300–350 g) was performed. Confirmation of pregnancy was accomplished by observing the presence of sperm in vaginal smears and designated as pregnancy day 1.

### Surgical procedures

All surgical stereotaxic procedures were performed under isoflurane anaesthesia and semi-sterile conditions on pregnancy day (PD) 14, lactation day (LD) 2 and in age-matched virgin rats. Following surgery, rats received a subcutaneous (s.c.) injection of antibiotic (0.03 ml enrofloxacin; 100 mg/1 ml Baytril, Bayer). Animals were allowed to recover before undergoing behavioural or MEK1/2 – ERK1/2 phosphorylation assessment six days after surgery (i.e. PD 20 or LD 8). Rats were housed singly after surgery and handled daily to habituate them to the respective central infusion procedure and to avoid non-specific stress responses during the experiment.

For analysis of the hypothalamic MAPK pathway in virgin, pregnant and lactating rats under the influence of icv vehicle, OXT (Sigma), or MEK1/2 inhibitor (U0126, Sigma) infusion, an indwelling icv guide cannula (stainless steel, 21 G, 12 mm long) was stereotaxically implanted 2 mm above the right lateral ventricle (AP: −1.0 mm bregma, ML: +1.6 mm lateral, DV: +1.8 mm below the surface of the skull; [43]) as previously described [3], [44].

For analysis of local effects of OXT within the PVN on anxiety-related behaviour, indwelling bilateral guide cannulas (stainless steel, 23 G, 12 mm long) were implanted 2 mm above both the left and right PVN (AP: −1.4 mm bregma, ML: −1.8 and +2.1 mm lateral; DV: +6 mm below the surface of the skull; angle 10°; [3], [43], and anchored to two stainless-steel screws using dental acrylic. The guide cannulas were kept viable with dummy cannulas, which were removed daily and cleaned during the handling procedure.

### Experimental protocols

#### Experiment 1. Effect of intra-PVN infusion of OXT on anxiety-related behaviour in virgin and lactating rats, and effects of local pre-treatment with the MEK1/2 inhibitor, U0126

The following experiment was performed in order to (i) compare the effects of OXT infused bilaterally into the PVN on anxiety-related behaviour in virgin and lactating rats and (ii) test for a possible involvement of the MEK1/2 – ERK1/2 cascade in the anxiolytic effect of both, OXT and lactation. Thus, conscious virgin and lactating rats received two subsequent bilateral PVN infusions. They were pre-treated with either vehicle (0.5 µl; 10% DMSO in Ringer solution, pH 7.4, Braun) or the MEK inhibitor U0126 (0.5 nmol/0.5 µl) bilaterally within the PVN 5 min prior to infusion of either vehicle, or OXT (0.01 nmol/0.5 µl) to assess four different groups (vehicle/vehicle, vehicle/OXT, U0126/vehicle or U0126/OXT). After each infusion, the cannulas were kept in place for 30 s to allow local substance diffusion. Anxiety-related behaviour was assessed in the light-dark box (LDB) 10 min later. The doses were chosen based on previous studies [3].

#### Experiment 2. Determination of basal and OXT-induced MEK1/2 and ERK1/2 activation within the hypothalamus of virgin, pregnant, and lactating rats

To assess the impact of reproductive status under basal and acute icv OXT conditions on hypothalamic MEK1/2 and ERK1/2 activation, conscious well-handled virgin, pregnant (PD20), and lactating (LD8) rats received either an acute vehicle (5 µl; Ringer solution, pH 7.4, Braun) or OXT (1 nmol) infusion. After icv infusion, the cannulas were kept in place for 30 s to allow local substance diffusion. Ten minutes later, rats were decapitated and the hypothalami dissected, immediately frozen, and stored at −20°C until required. The hypothalamus was dissected rostrally at the level of the optic chiasm, and caudally through the median eminence. Laterally, the lateral ventricle was used as a landmark to remove the most lateral parts of the hypothalamus. Dorsally, the third ventricle represented the top most level of the PVN-enriched region. The suprachiasmatic and remaining part of the supraoptic nuclei were also removed. This results in a hypothalamic tissue block containing mostly the PVN with some bed nucleus of the stria terminalis, medial preoptic area, and the anterior hypothalamic nuclei (Fig. 1 and [43]).

#### Experiment 3. Effects of blockade of MEK1/2 activity on basal and OXT-induced ERK1/2 activation within the hypothalamus

To assess if icv pre-treatment with the MEK1/2 kinase inhibitor, U0126, reduced basal or OXT-induced ERK1/2 activation in virgin or lactating (LD8) rats, the following groups were compared: vehicle/vehicle, U0126 (1 nmol)/vehicle, vehicle/OXT (1 nmol) or U0126/OXT. The icv infusions were 10 min apart, and 10 min after the second infusion, the hypothalami were isolated as described in Experiment 2.

### Light-dark box

To assess the effects of OXT and MEK inhibitor infusions bilaterally in the PVN of virgin or lactating rats on anxiety-related behaviour, the animals were tested in the LDB seven days after the implantation of the guide cannulas (i.e. LD 8 or equivalent in age-matched virgins) and 10 min after the last drug infusion. The LDB test was performed as previously described [22], [44]. Briefly, the LDB setup consisted of two compartments; one lit compartment (40×50 cm, 350 lux; light box) and one dark compartment (40×30 cm, 70 lux). The floors in each compartment were divided into squares (10×10 cm) and the compartments were connected via a small opening (7.5×7.5 cm) enabling transition between the compartments. Rats were placed in the dark compartment and line-crosses, time spent in each compartment, rearings, and latency to first light compartment entry during the 5-min test, were assessed on-line via a camera located above the box. The time spent in the light box by the vehicle/vehicle group was set to 100% for each experiment.

### Western blot analysis of protein phosphorylation

Cytoplasmatic and nuclear proteins were extracted using a protein extraction kit (Active Motif, Rixensart, Belgium). Briefly, single hypothalamic tissue blocks were homogenised in 300 µl hypotonic buffer (supplemented with 0.1 mM DTT, 0.1 mM detergent, phosphatase and protease inhibitors as included in the kit), and incubated on ice for 15 min. Following centrifugation (10 min, 850 g, 4°C), the supernatant containing cytoplasmatic proteins was collected. To ensure complete lysis, the pellet was resuspended in 200 µl of hypotonic buffer (supplemented only with phosphatase and protease inhibitors) and incubated on ice for 15 min. Then 50 µl/ml of detergent was added, the mixture vigorously vortexed and centrifuged (3 min, 14,000 g, 4°C). The supernatant was collected and pooled with the supernatant collected earlier to an end volume of 500 µl. The pellet was washed once with ice-cold phosphate-buffered saline (PBS), to wash away any remaining cytoplasmatic proteins, and resuspended in 100 µl complete lysis buffer, supplemented with DTT, and phosphatase and protease inhibitors, as indicated by the manufacturer. The samples were incubated on ice for 30 min, then vortexed, centrifuged (10 min, 14,000 g, 4°C), and supernatants containing nuclear proteins collected. The protein concentrations were determined using the Pierce BCA Protein Assay Kit (Thermo Scientific, Rockford, USA).

Thirty micrograms of each protein extract were separated on a 12,5% sodium dodecyl sulphate-polyacrylamide gel and transferred onto a nitrocellulose membrane (Bio-Rad). Non-specific binding was blocked in Tris-buffered saline/0.1% Tween-20 (TBST, pH 7.6) supplemented with 5% bovine serum albumin (BSA, Sigma) overnight at 4°C. Next, the blots were incubated with a specific antibody against phosphorylated ERK1/2 or MEK1/2 (1∶1,000 each; Cell Signaling Technology, nrs 9101 and 9154, respectively). After 5 hours incubation at room temperature, the blots were washed extensively with TBST, and incubated with peroxidase-conjugated donkey anti-rabbit IgG (1∶1,000; Amersham, Little Chalfont, England) for 30 min. Then the blots were washed again, and probed with an anti-β-tubulin (1∶1,000, Cell Signaling Technology), GAPDH, or TATA box binding-protein (TBP) antibody (both 1∶1,000; Abcam) as total protein loading controls; β-tubulin and GAPDH for each cytoplasmic fraction, and TBP for each nuclear fraction. After incubation overnight at 4°C, the blots were washed and treated with secondary antibody as described above. Bands were visualised using ECL western blot detection reagents (GE Healthcare, Little Chalfont, UK), and images were acquired with the ChemiDoc XRS+ system (Bio-Rad). After imaging, immunocomplexes were removed from the blot with Re-Blot Plus Solution (Millipore), probed with anti-total ERK1/2 or MEK1/2 antibodies (1∶1,000; Cell Signaling Technology, nrs 9102 and 9122, respectively), and imaged as described above to control for total amount of kinases loaded on the gel.

### Verification of cannula placements

After the experimental procedure, the animals were sacrificed. For verification of the placement of icv cannula, following dissection of the hypothalamus, the rest of the brain was snap-frozen in isopentane cooled to −32°C by dry-ice. Localisation of the cannula tract was then performed using 40-µm cryosections stained with Nissl and assisted with a brain atlas [43]. For PVN cannula verification, blue dye was infused into the PVN as described above, then the brain was snap-frozen in isopentane and histological assessment performed as described for the icv cannulas above. Only animals with correctly positioned cannulas were included in the statistical analyses.

### Statistical analyses

Signalling and behavioural data were analysed using either a one-way or a two-way analysis of variance (ANOVA, factors reproductive state x treatment, factors treatment 1× treatment 2). Behavioural data is expressed in comparison to vehicle-treated groups of both virgins and lactating dams, as the experiments were performed separately. Any overall statistical differences, which were set at P<0.05, were further analysed using a Fisher's *post-hoc* test. Separate non-parametric Mann-Whitney U tests (MWU) were performed. Data are expressed as group mean ± S.E.M. Statistical analyses were performed using SPSS for Windows (version 16; SPSS Inc, Chicago, IL, USA).

## References

[1] Davis S, Laroche S (2006). Mitogen-activated protein kinase/extracellular regulated kinase signalling and memory stabilization: a review.. Genes Brain Behav.

[2] Satoh Y, Endo S, Nakata T, Kobayashi Y, Yamada K (2011). ERK2 contributes to the control of social behaviors in mice.. J Neurosci.

[3] Blume A, Bosch OJ, Miklos S, Torner L, Wales L (2008). Oxytocin reduces anxiety via ERK1/2 activation: local effect within the rat hypothalamic paraventricular nucleus.. Eur J Neurosci.

[4] Chen J, Volpi S, Aguilera G (2008). Anti-apoptotic actions of vasopressin in H32 neurons involve MAP kinase transactivation and Bad phosphorylation.. Exp Neurol.

[5] Landgraf R, Neumann ID (2004). Vasopressin and oxytocin release within the brain: a dynamic concept of multiple and variable modes of neuropeptide communication.. Front Neuroendocrinol.

[6] Bunck M, Czibere L, Horvath C, Graf C, Frank E (2009). A hypomorphic vasopressin allele prevents anxiety-related behavior.. PLoS One.

[7] Murgatroyd C, Wigger A, Frank E, Singewald N, Bunck M (2004). Impaired repression at a vasopressin promoter polymorphism underlies overexpression of vasopressin in a rat model of trait anxiety.. J Neurosci.

[8] Brunton PJ, Russell JA (2008). The expectant brain: adapting for motherhood.. Nat Rev Neurosci.

[9] Hillerer KM, Neumann ID, Slattery DA (2011). From Stress to Postpartum Mood and Anxiety Disorders: How Chronic Peripartum Stress Can Impair Maternal Adaptations..

[10] Slattery DA, Neumann ID (2008). No stress please! Mechanisms of stress hyporesponsiveness of the maternal brain.. J Physiol.

[11] Carter CS, Altemus M, Chrousos GP (2001). Neuroendocrine and emotional changes in the post-partum period.. Prog Brain Res.

[12] Windle RJ, Kershaw YM, Shanks N, Wood SA, Lightman SL (2004). Oxytocin attenuates stress-induced c-fos mRNA expression in specific forebrain regions associated with modulation of hypothalamo-pituitary-adrenal activity.. J Neurosci.

[13] Tomizawa K, Iga N, Lu YF, Moriwaki A, Matsushita M (2003). Oxytocin improves long-lasting spatial memory during motherhood through MAP kinase cascade.. Nat Neurosci.

[14] Kosfeld M, Heinrichs M, Zak PJ, Fischbacher U, Fehr E (2005). Oxytocin increases trust in humans.. Nature.

[15] Labuschagne I, Phan KL, Wood A, Angstadt M, Chua P (2010). Oxytocin attenuates amygdala reactivity to fear in generalized social anxiety disorder.. Neuropsychopharmacology.

[16] Meyer-Lindenberg A, Domes G, Kirsch P, Heinrichs M (2011). Oxytocin and vasopressin in the human brain: social neuropeptides for translational medicine.. Nat Rev Neurosci.

[17] Viviani D, Charlet A, van den Burg E, Robinet C, Hurni N (2011). Oxytocin selectively gates fear responses through distinct outputs from the central amygdala.. Science.

[18] Neumann ID, Torner L, Wigger A (2000). Brain oxytocin: differential inhibition of neuroendocrine stress responses and anxiety-related behaviour in virgin, pregnant and lactating rats.. Neuroscience.

[19] Windle RJ, Shanks N, Lightman SL, Ingram CD (1997). Central oxytocin administration reduces stress-induced corticosterone release and anxiety behavior in rats.. Endocrinology.

[20] Bosch OJ, Meddle SL, Beiderbeck DI, Douglas AJ, Neumann ID (2005). Brain oxytocin correlates with maternal aggression: link to anxiety.. J Neurosci.

[21] Neumann I, Ludwig M, Engelmann M, Pittman QJ, Landgraf R (1993). Simultaneous microdialysis in blood and brain: oxytocin and vasopressin release in response to central and peripheral osmotic stimulation and suckling in the rat.. Neuroendocrinology.

[22] Waldherr M, Neumann ID (2007). Centrally released oxytocin mediates mating-induced anxiolysis in male rats.. Proc Natl Acad Sci U S A.

[23] Landgraf R, Kessler MS, Bunck M, Murgatroyd C, Spengler D (2007). Candidate genes of anxiety-related behavior in HAB/LAB rats and mice: focus on vasopressin and glyoxalase-I.. Neurosci Biobehav Rev.

[24] Strakova Z, Copland JA, Lolait SJ, Soloff MS (1998). ERK2 mediates oxytocin-stimulated PGE2 synthesis.. Am J Physiol.

[25] Zhong M, Yang M, Sanborn BM (2003). Extracellular signal-regulated kinase 1/2 activation by myometrial oxytocin receptor involves Galpha(q)Gbetagamma and epidermal growth factor receptor tyrosine kinase activation.. Endocrinology.

[26] Bale TL, Davis AM, Auger AP, Dorsa DM, McCarthy MM (2001). CNS region-specific oxytocin receptor expression: importance in regulation of anxiety and sex behavior.. J Neurosci.

[27] Jungling K, Seidenbecher T, Sosulina L, Lesting J, Sangha S (2008). Neuropeptide S-mediated control of fear expression and extinction: role of intercalated GABAergic neurons in the amygdala.. Neuron.

[28] Tehovnik EJ, Sommer MA (1997). Effective spread and timecourse of neural inactivation caused by lidocaine injection in monkey cerebral cortex.. J Neurosci Methods.

[29] Stewart S, Sundaram M, Zhang Y, Lee J, Han M (1999). Kinase suppressor of Ras forms a multiprotein signaling complex and modulates MEK localization.. Mol Cell Biol.

[30] Pullikuth A, McKinnon E, Schaeffer HJ, Catling AD (2005). The MEK1 scaffolding protein MP1 regulates cell spreading by integrating PAK1 and Rho signals.. Mol Cell Biol.

[31] Maiga O, Philippe M, Kotelevets L, Chastre E, Benadda S (2011). Identification of mitogen-activated protein/extracellular signal-responsive kinase kinase 2 as a novel partner of the scaffolding protein human homolog of disc-large.. Febs J.

[32] Brandt KJ, Carpintero R, Gruaz L, Molnarfi N, Burger D (2010). A novel MEK2/PI3Kdelta pathway controls the expression of IL-1 receptor antagonist in IFN-beta-activated human monocytes.. J Leukoc Biol.

[33] Chen G, Ghosh P, Longo DL (2011). Distinctive mechanism for sustained TGF-beta signaling and growth inhibition: MEK1 activation-dependent stabilization of type II TGF-beta receptors.. Mol Cancer Res.

[34] Lin HP, Chang JY, Lin SR, Lee MH, Huang SS (2011). Identification of an In Vivo MEK/WOX1 Complex as a Master Switch for Apoptosis in T Cell Leukemia.. Genes Cancer.

[35] Jo C, Cho SJ, Jo SA (2011). Mitogen-activated protein kinase kinase 1 (MEK1) stabilizes MyoD through direct phosphorylation at tyrosine 156 during myogenic differentiation.. J Biol Chem.

[36] Burgermeister E, Chuderland D, Hanoch T, Meyer M, Liscovitch M (2007). Interaction with MEK causes nuclear export and downregulation of peroxisome proliferator-activated receptor gamma.. Mol Cell Biol.

[37] Oliet SH (2002). Functional consequences of morphological neuroglial changes in the magnocellular nuclei of the hypothalamus.. J Neuroendocrinol.

[38] Theodosis DT, Poulain DA (1989). Neuronal-glial and synaptic plasticity in the adult rat paraventricular nucleus.. Brain Res.

[39] Theodosis DT (2002). Oxytocin-secreting neurons: A physiological model of morphological neuronal and glial plasticity in the adult hypothalamus.. Front Neuroendocrinol.

[40] Satoh Y, Endo S, Ikeda T, Yamada K, Ito M (2007). Extracellular signal-regulated kinase 2 (ERK2) knockdown mice show deficits in long-term memory; ERK2 has a specific function in learning and memory.. J Neurosci.

[41] Selcher JC, Nekrasova T, Paylor R, Landreth GE, Sweatt JD (2001). Mice lacking the ERK1 isoform of MAP kinase are unimpaired in emotional learning.. Learn Mem.

[42] Mazzucchelli C, Vantaggiato C, Ciamei A, Fasano S, Pakhotin P (2002). Knockout of ERK1 MAP kinase enhances synaptic plasticity in the striatum and facilitates striatal-mediated learning and memory.. Neuron.

[43] Paxinos G, Watson C (1998). The Rat Brain in Sterotaxic Coordinates (4th edition)..

[44] Slattery DA, Neumann ID (2010). Chronic icv oxytocin attenuates the pathological high anxiety state of selectively bred Wistar rats.. Neuropharmacology.

